# Naringenin Promotes Gastrointestinal Motility in Mice by Impacting the SCF/c-Kit Pathway and Gut Microbiota

**DOI:** 10.3390/foods13162520

**Published:** 2024-08-12

**Authors:** Lei Wu, Yao Niu, Boyang Ren, Shengyu Wang, Yuhong Song, Xingyu Wang, Kai Zhao, Zhao Yue, Yaru Li, Jianhua Gao

**Affiliations:** College of Life Sciences, Shanxi Agricultural University, Taigu 030801, China; wulei@sxau.edu.cn (L.W.); n248199@163.com (Y.N.); 17835732081@163.com (B.R.); 13233451939@163.com (S.W.); syh17835876918@163.com (Y.S.); 19834544330@163.com (X.W.); zhao13935073377@163.com (K.Z.); yz16635424967@163.com (Z.Y.); lyr1874654346@163.com (Y.L.)

**Keywords:** naringenin, gastrointestinal motility, gastrointestinal hormones, gut microbiota

## Abstract

Naringenin (NRG) is widely found in citrus fruits and has anti-inflammatory, hypoglycemic, and immunomodulatory effects. Previous studies have shown that NRG promotes gastrointestinal motility in mice constipation models, but there are few systematic evaluations of its effects on normal animals. This study first clarified the promotive effects of NRG on gastric emptying and small intestine propulsion (*p* < 0.01). NRG can also regulate the release of gastrointestinal hormones, including enhancing gastrin (GAS) and motilin (MTL) (*p* < 0.01), while reducing vasoactive intestinal peptide (VIP) secretion (*p* < 0.01). Using NRG to stimulate the isolated stomach, duodenum, and colon showed similar promotive effects to those observed in vivo (*p* < 0.01). A Western blot analysis indicated that this effect may be mediated by increasing the expression of stem cell factor (SCF) and its receptor (c-Kit) in these three segments, thus regulating their downstream pathways. It is worth noting that NRG can also increase the proportion of beneficial bacteria (Planococcaceae, *Bacteroides acidifaciens*, *Clostridia_UCG-014*) in the intestine and reduce the quantity of harmful bacteria (*Staphylococcus*). These findings provide a new basis for the application of NRG.

## 1. Introduction

Naringenin (NRG) is a flavanone compound predominantly encountered in its glycosidic form, known as naringin, which is abundant in citrus fruits like oranges and grapefruits, as well as in pomelos, tomatoes, and a variety of other fruit types. Although flavanones constitute a relatively minor proportion of plant compounds, their significance in the human diet is amplified owing to the high consumption rates of citrus fruits and their derived products, positioning them as a notable contributor to overall flavonoid intake.

NRG has numerous beneficial effects on the human body, including controlling metabolic diseases, strengthening the body’s antioxidant defenses, modulating immune system activities, and providing both anti-inflammatory and anti-atherosclerotic benefits [1,2,3]. These actions highlight NRG’s potential in promoting overall health and preventing various metabolic disorders. In dietary studies, particularly with mice lacking LDL receptors on high-fat diets, which, as a result, suffered from lipid metabolism disorders, insulin and glucose intolerance, and obesity, NRG has shown promising results. It has enhanced liver cell sensitivity to insulin and improved the cellular response to low doses of insulin. This suggests that NRG can help rectify metabolic issues tied to insulin resistance, offering a natural approach to managing conditions such as type 2 diabetes and metabolic syndrome [4,5]. Additionally, NRG has demonstrated strong inhibitory effects on the growth of C6 glioma cells in rat brains by reducing the levels of several important molecules, including protein kinase C, nuclear factor kappa B (NF-κB), cyclin D1, and cyclin-dependent kinase 4 (CDK4), as well as by mitigating oxidative stress [6].

In previous studies, the absorption mechanisms, bioavailability, and metabolic pathways of NRG within the gastrointestinal tract have been reported [7,8]. Flavonoids generally exhibit low bioavailability within the human body, a characteristic shared by NRG, which demonstrates an oral bioavailability of approximately 15% [9]. The absorption of NRG is facilitated by both passive diffusion and active transport mechanisms, and these processes are notably independent of pH conditions [10]. The absorption efficiency of NRG varies across distinct segments of the gastrointestinal tract. For example, in one previous study, the absorption rate was approximately 47% in the duodenum, 42% in the jejunum, and 39% in the ileum [11]. Furthermore, Rebello et al. conducted a comprehensive investigation into the pharmacokinetic parameters of NRG. In adult humans, the permissible daily intake of NRG is established to range from 150 to 900 mg, with its serum concentrations exhibiting a direct proportionality to the administered dosages. Notably, in vivo maximum concentrations of 15.76 ± 7.88 μM for a 150 mg dose and 48.45 ± 7.88 μM for a 600 mg dose were recorded approximately 3.17 ± 0.74 h and 2.41 ± 0.74 h post-administration, respectively. The pharmacokinetic profile of NRG has revealed a half-life of 3.0 h for a 150 mg dose and 2.65 h for a 600 mg dose [12]. In the circulatory systems of rats, NRG predominantly exists in conjugated forms, namely, sulfate and glucuronide [13]. It has been demonstrated that NRG can ameliorate constipation symptoms induced by loperamide in mice through the modulation of the secretion of various gastrointestinal hormones and the regulation of related signaling pathways [14].

However, the precise effects of NRG on the gastrointestinal tract in normative animals following ingestion remains to be elucidated. Therefore, the aim of this research was to explore the effects of NRG on gastrointestinal motility in mice and to shed light on the underlying mechanisms by analyzing alterations in gastrointestinal hormone secretion, the canonical ligand/receptor tyrosine kinase signaling pathway (the stem cell factor (SCF) and its receptor (tyrosine protein kinase Kit, c-Kit)), which are responsible for smooth muscle contraction, as well as analyzing the composition of the gut microbiota.

## 2. Materials and Methods

### 2.1. Drugs

NRG was purchased from Beijing Solarbio Science & Technology Co., Ltd. (catalog number SN8020, purity ≥ 98% by HPLC analysis, Beijing, China).

### 2.2. Animal Grouping, Drug Administration, and Sample Collection

Forty-two specific-pathogen-free (SPF) Kunming mice (both female and male), aged 8 weeks and weighing 20–22 g, were obtained from Spelford Biotechnology Co., Ltd. (Beijing, China). The mice were given clean water and specific animal maintenance feed daily and maintained under a regulated 12 h light/dark cycle. Subsequent to acclimatization, the mice were randomly divided into five groups for gavage, as follows: a negative control group (NC, saline), a positive control group (Mosapride at 1 mg/kg), a high-concentration NRG group (NRG_H, 25 mg/mL), a medium-concentration NRG group (NRG_M, 12.5 mg/mL), a the low-concentration NRG group (NRG_L, 6.25 mg/mL). To facilitate dissolution, dimethyl sulfoxide (DMSO) at 0.1% was incorporated into each preparation, and the mice were subjected to gavage over a period of 7 days. On the final day of administration, the mice were anesthetized. Following the completion of gastric emptying and small intestinal propulsion assessments, samples of stomach, duodenum, and colon tissues measuring 5 × 5 mm were harvested from each group of euthanized mice. These tissue samples were then thrice washed with physiological saline to expunge any intestinal content and subsequently preserved at −80 °C for future Western blot analysis. Additionally, cecal contents were gathered into 1 mL sterile centrifuge tubes, promptly frozen at −80 °C, and transported on dry ice for further analysis. All procedures involving animals were conducted with the approval of the Experimental Animal Ethics Committee of Shanxi Agricultural University (Taigu, China), and they adhered strictly to the regulations and guidelines set forth by the same committee. The ethical approval for this study was granted under the permit number SXAU-EAW-2023SK.R.P.012011194.

### 2.3. Gastric Emptying and Small Intestinal Propulsion Assessments

On day six of continuous gavage, the mice had been fasting without water for 18 h. Drawing upon established methodologies in the literature [15], with specific modifications, a semi-solid marker was prepared. The recipe entailed dissolving 5 g of sodium carboxymethyl cellulose in 125 mL of distilled water, followed by the addition of 8 g of milk powder, 4 g of glucose, 4 g of starch, and 1 g of activated charcoal powder. This mixture was thoroughly combined to yield 150 mL of a semi-solid mixture which was subsequently refrigerated and equilibrated to 20 °C prior to administration. On the seventh day, subsequent to the completion of the dosing regimen, each mouse was administered 0.8 mL of the semi-solid mixture. The precise weight of the administered paste (M_p_) was meticulously measured and documented. Approximately 40 min following the administration, the animals were sedated with pentobarbital sodium at a dosage of 50 mg/kg. Blood samples were collected through cardiac punctures in the mice. Following sacrifice, the full stomach weight (M_fs_), the net stomach weight (M_ns_), the entire length from the pylorus to the cecum (L_t_), and the distance of carbon propulsion (L_c_) within the small intestine were individually assessed. The rates of gastric emptying (Equation (1)) and the propulsion speed in the small intestine (Equation (2)) were determined by applying relevant mathematical equations. Equations (1) and (2) have been described in a previous report [16].

### 2.4. Quantitative Assessment of the Gastrointestinal Hormone Concentrations in the Murine Serum

The blood samples gathered according to the protocol outlined in Section 2.3 underwent centrifugation at a force of 550× *g* for 15 min at a temperature of 4 °C, which facilitated the isolation of the serum. The isolated serum was then transferred into RNase-free, autoclaved cryovials and subsequently preserved at a temperature of −80 °C for subsequent examination. The serum levels of gastrin (GAS, catalog no. D731177), vasoactive intestinal peptide (VIP, catalog no. D721193) (both acquired from Sangon Biotech Co., Ltd. (Shanghai, China)), and motilin (MTL, catalog no. H182-1-1) (sourced from NanJing JianCheng Bioengineering Institute (Nanjing, China)) were quantified by employing the double-antibody sandwich enzyme-linked immunosorbent assay (ELISA) technique. All assays were conducted in strict accordance with the protocols provided by the respective manufacturers.

### 2.5. Spontaneous Activity of the Isolated Gastrointestinal Tissues

This investigation was carried out in accordance with the method described in our prior study [16], though with modifications. Twelve mice had been fasting without water for 24 h, and then the animals were anesthetized to isolate the muscle strips of their stomachs, duodenums, and colons. Eighty microliters of NRG_H (H) was introduced into the Tyrode solution to serve as the treatment condition.

### 2.6. Western Blot Analysis

The stomach, duodenum, and colon tissues from the murine specimens were accurately weighed and subjected to pulverization in liquid nitrogen, and then they were treated with RIPA buffer (catalog no. C5029, Beijing Biosynthesis Biotechnology Co., Ltd., Beijing, China), which included 1% of both protease and phosphatase inhibitors. The resulting homogenates were kept on ice for 30 min to promote cell lysis. Centrifugation was then performed at 4 °C and 14,000 rpm for 10 min, leading to the collection of the supernatants containing the protein extracts. Protein concentrations within the extracts were determined by a bicinchoninic acid (BCA) protein assay. Next, 10 μg of each protein sample was loaded onto sodium dodecyl sulfate-polyacrylamide gel electrophoresis (SDS-PAGE) for separation and then transferred to polyvinylidene difluoride (PVDF) membranes via electrotransfer. The membranes were incubated in TBST buffer (catalog no. T1081, Beijing Solarbio Science & Technology Co., Ltd.) supplemented with 5% non-fat dried milk for 1.5 h to achieve blocking. Overnight incubation at 4 °C was then conducted with the primary antibodies against SCF (catalog no. bs-0545R, Beijing Biosynthesis Biotechnology Co., Ltd.) or a c-Kit (catalog no. bsm-52036R, Beijing Biosynthesis Biotechnology Co., Ltd.) at a dilution of 1:1000. After incubation, the membranes were washed thrice with TBST. HRP-conjugated Affinipure Goat Anti-Rabbit IgG (H + L) secondary antibodies were subsequently added at a 1:5000 dilution and allowed to incubate at room temperature for 1 h. Then, the membranes underwent an additional three washes with TBST buffer. For visualizing the protein bands, the membranes were developed and then placed onto an exposure board within the ChemiDoc MP Imaging System (Bio-Rad Laboratories Inc., Hercules, CA, USA) for image acquisition in a darkroom setting. GAPDH served as the loading control throughout the experiment.

### 2.7. 16S rDNA Sequencing

The primary procedures encompassing DNA extraction, PCR amplification and sequencing of the 16S rRNA gene, Illumina Miseq sequencing, and data processing have been previously delineated [17]. In summary, the total microbial DNA was isolated from the mouse fecal specimens utilizing an E.Z.N.A.^®^ Soil DNA Kit (Omega Bio-Tek, Norcross, GA, USA) in accordance with the manufacturer’s guidelines. The integrity of the isolated DNA was evaluated by agarose gel electrophoresis. A NanoDrop 2000 spectrophotometer (Thermo Fisher Scientific, Chicago, IL, USA) was used to determine the concentrations and purity of the DNA samples. The V3–V4 variable regions of the 16S rRNA gene were amplified through PCR (ABI GeneAmp^®^ 9700, ABI, Carlsbad, CA, USA) employing the primer sequences 338F (5′-ACTCCTACGGGAGGCAGCAG-3′) and 806R (5′-GGACTACHVGGGTWTCTAAT-3′). The PCR products were purified with an AxyPrep DNA Gel Extraction Kit (Axygen, Union City, CA, USA). Quantification of the purified DNA was conducted using a Quantus™ Fluorometer (Promega, Madison, WI, USA). The sequencing libraries were constructed using a NEXTFLEX Rapid DNA-Seq Kit (Bioo Scientific, Austin, TX, USA) and subsequently sequenced on an Illumina MiSeq PE300 platform (Majorbio Bio-Pharm Technology Co., Ltd., Shanghai, China). The raw sequence data were then submitted to the NCBI SRA database (accession no. PRJNA1114870). The operational taxonomic unit (OTU) clustering was conducted with the UPARSE software package (version 7.1).

### 2.8. Data Analysis

The data were represented as means ± SEMs. Statistical comparisons were carried out using one-way analysis of variance (ANOVA) with SPSS (version 26.0, International Business Machines Corporation, Armonk, NY, USA). Data visualization was performed with GraphPad Prism (version 9.0, GraphPad Software Inc., Boston, MA, USA).

## 3. Results

### 3.1. NRG Enhances Gastric Emptying and Small Intestinal Propulsion in Normal Mice

**Figure 1:**
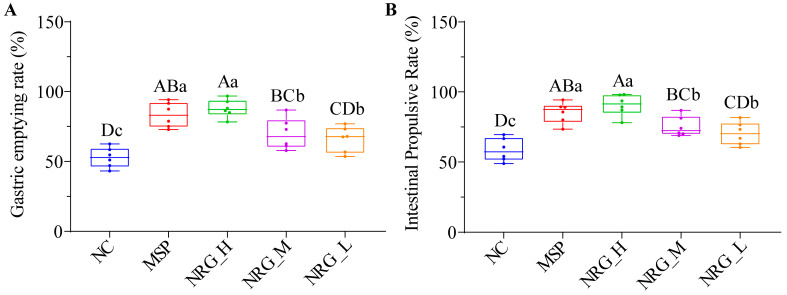
Impact of varying concentrations of NRG on gastric emptying rate (**A**) and intestinal propulsive rate (**B**) in vivo. Statistical significance between the groups is indicated by the capital letters (*p* < 0.01) and/or lowercase letters (*p* < 0.05) above the bars. Each experimental group consisted of six mice.

In this study, we first examined the potential impact of NRG on gastric emptying and intestinal motility in normal mice. It was observed that all three concentrations of NRG facilitated enhanced gastric emptying (Figure 1A, *p* < 0.01) as well as increased intestinal transit (Figure 1B, *p* < 0.01). Notably, the efficacy of the high concentrations of NRG (NRG_H) was found to be statistically indistinguishable from that of the 5-HT_4_ receptor agonist (MSP) (*p* > 0.05), whereas the medium (NRG_M) and low concentrations (NRG_L) demonstrated significantly reduced effects when compared to the MSP (*p* < 0.05).

### 3.2. NRG Regulates Three Gastrointestinal Hormone Expression Levels in Serum

**Figure 2:**
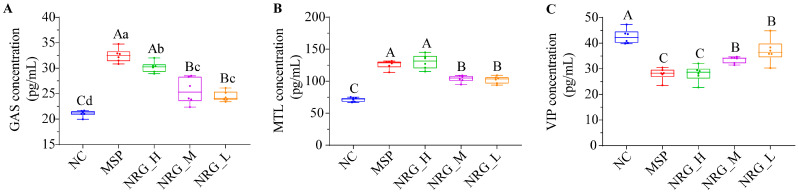
Impact of varying concentrations of NRG on the secretion of GAS (**A**), MTL (**B**), and VIP (**C**) in mouse serum after 7 d of administration. Statistical significance between the groups is indicated by the capital letters (*p* < 0.01) and lowercase letters (*p* < 0.05) above the bars. Each experimental group consisted of six mice.

The tested concentrations of NRG markedly enhanced the serum secretion levels of GAS and MTL (Figure 2A,B, *p* < 0.01) while concurrently decreasing the secretion levels of VIP (Figure 2C, *p* < 0.01). Notably, NRG_H demonstrated an impact on the MTL and VIP secretions that was analogous to that of the MSP, although it exhibited a marginally reduced effect on GAS secretion (*p* < 0.05). NRG_M and NRG_L yielded similar outcomes, with both being significantly less potent than NRG_H in their effects (*p* < 0.01).

### 3.3. NRG Boosts the Spontaneous Activity of Isolated Gastrointestinal Tissues

**Figure 3:**
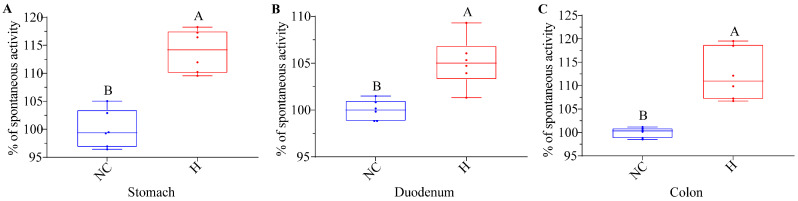
In vitro effects of high NRG concentrations on the contraction of isolated muscle strips from various gastrointestinal tissues (**A**) Stomach; (**B**) Duodenum; (**C**) Colon. Contraction activity is presented as a percentage (%) of the spontaneous activity relative to the negative control (NC, normalized to 100%). Statistical significance between the groups is indicated by the capital letters (*p* < 0.01) above the bars. Each experimental group consisted of six mice.

To elucidate the precise site of action of NRG within the gastrointestinal tract, muscle strips isolated from the stomachs, duodenums, and colons of the mice were separately incubated with high concentrations of NRG. The findings revealed a statistically significant augmentation in muscle contractility across all tested segments (Figure 3, *p* < 0.01).

### 3.4. NRG Enhances the Expression of SCF and c-Kit Proteins in Gastrointestinal Tissues

**Figure 4:**
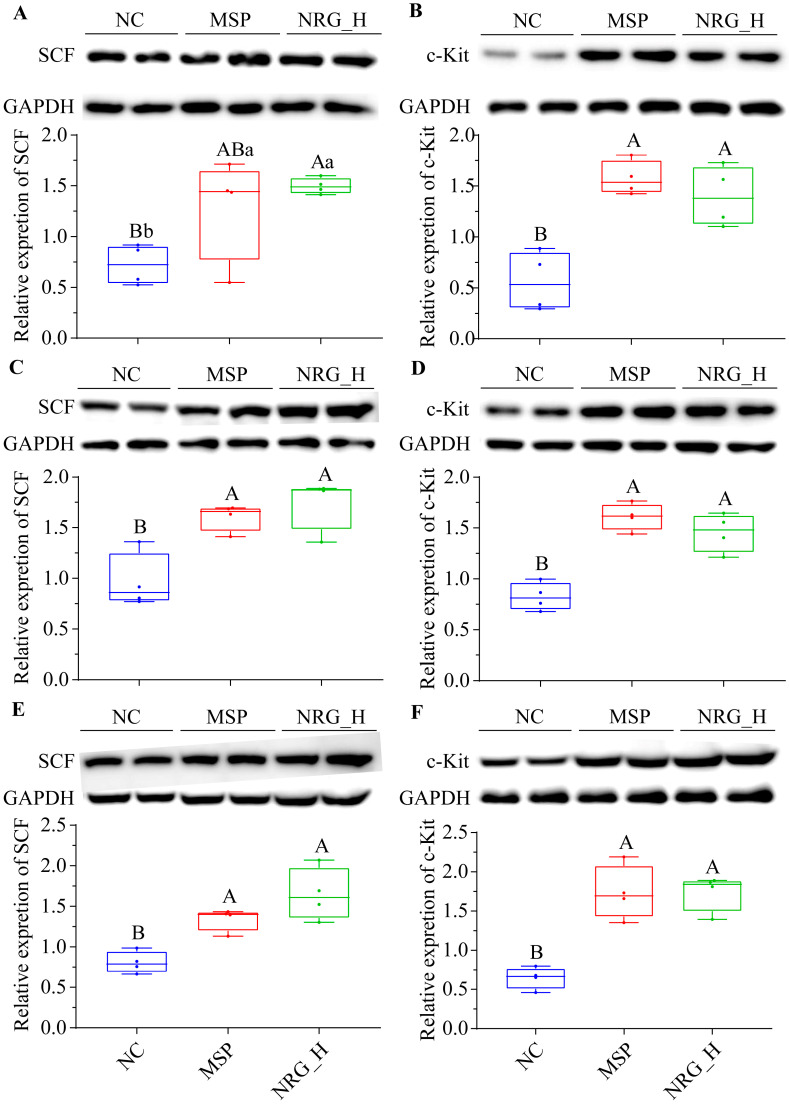
Western blotting analysis of SCF and c-Kit in the stomach (**A**,**B**), duodenum (**C**,**D**), and colon (**E**,**F**) tissues of each group. Statistical significance between the groups is indicated by the capital letters (*p* < 0.01) and/or lowercase letters (*p* < 0.05) above the bars. Each experimental group consisted of four mice.

Compared to the NC group, following a one-week administration period, the NRG_H group exhibited a statistically significant elevation in SCF and c-Kit protein expression within their stomachs (*p* < 0.05 and *p* < 0.01, respectively). The magnitude of these alterations paralleled those observed in the MSP group (Figure 4A,B). Similarly, in the duodenum and colon tissues, the changes in SCF and c-Kit protein expression mirrored those observed in the stomach tissues (Figure 4C–F).

### 3.5. NRG Modulates Gut Microbiota Composition

**Figure 5:**
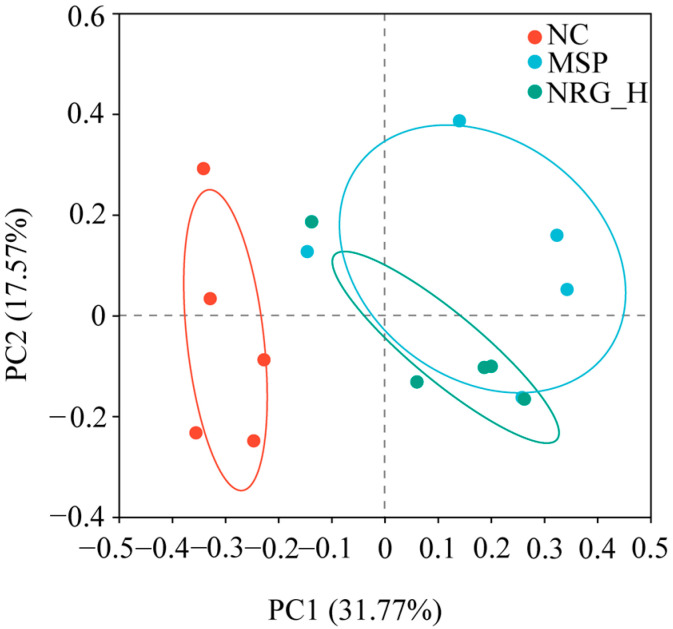
PCoA analysis of the OTU levels of gut microbiota. Each experimental group consisted of five mice.

Utilizing 16S rDNA sequencing, alterations in the gut microbiota composition across the various treatment groups post-drug administration were ascertained. Principal coordinates analysis (PCoA) demonstrated that the gut microbiota composition of the NC group markedly differed from those of the MSP and NRG_H groups. Conversely, the microbiota profiles within the MSP and NRG_H groups exhibited greater degrees of similarity (Figure 5).

**Figure 6:**
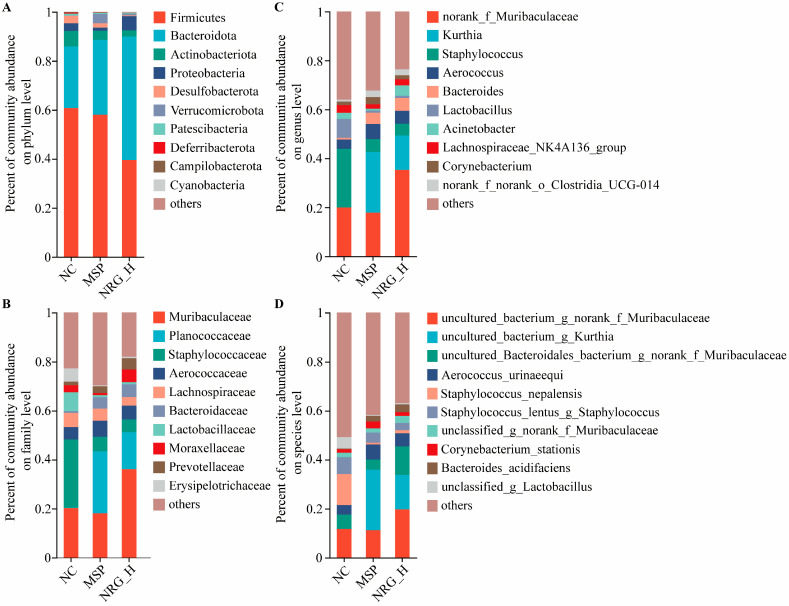
The variations in gut microbiota composition are displayed at the phylum (**A**), family (**B**), genus (**C**), and species levels (**D**), respectively. Each experimental group consisted of five mice.

At the phylum level, the predominant microbial groups identified were Firmicutes, Bacteroidetes, Actinobacteria, Proteobacteria, and Desulfobacterota, with Firmicutes and Bacteroidetes constituting the highest proportions. Specifically, in the NC, MSP, and NRG_H groups, the proportions of Firmicutes and Bacteroidetes were 60.76% and 25.16%, 58.03% and 30.56%, and 39.55% and 50.36%, respectively. This indicated that following the NRG_H treatment, the proportion of Bacteroidetes approximately doubled. Notably, Verrucomicrobiota demonstrated a marked elevation in abundance within the MSP group relative to the NC group, with an increase of 183.69%, but in the NRG_H group, only a 31.14% increment was observed. The abundance of Actinobacteriota and Desulfobacterota decreased after treatment, with the MSP group seeing reductions of 41.55% and 42.89% and the NRG group experiencing reductions of 59.22% and 90.28%, respectively (Figure 6A).

At the family level, the abundances of Muribaculaceae and Moraxellaceae in the MSP group experienced reductions of 10.44% and 70.41%, respectively, whereas in the NRG_H group, they increased by 78.59% and 76.15%, respectively. The family Planococcaceae demonstrated a significant post-administration increase, with abundances 147.76- and 77.41-fold higher in the MSP and NRG_H groups, respectively, compared to the NC group. Bacteroidaceae displayed analogous patterns, with their abundances in the MSP and NRG_H groups being 6.80- and 7.67-fold greater than in the NC group, respectively. Conversely, the abundances of Staphylococcaceae decreased post-administration, with reductions of 78.76% observed in the MSP group and 81.19% in the NRG_H group (Figure 6B).

At the genus level, following administration, the prevalence of *Kurthia* escalated from 0.03% in the NC group to 24.70% in the MSP group and 14.05% in the NRG_H group. Concurrently, *Bacteroides* followed a similar trajectory, rising from 0.68% in the NC group to 4.62% in the MSP group and 5.21% in the NRG_H group, respectively. The genus *Clostridia_UCG-014* also underwent significant changes, with its abundance reaching 2.65% in the MSP group and 2.43% in the NRG_H group, which were increases of 2.49 and 2.20-fold compared to the baseline of 0.76% in the NC group, respectively. Conversely, *Lactobacillus*, which constituted 7.70% in the NC group, experienced a marked decline post-administration, with reductions of 88.47% and 88.66% observed in the MSP and NRG_H groups, respectively (Figure 6C).

At the species level, *Bacteroides acidifaciens* demonstrated a pronounced increase following drug treatment, with abundances of 2.27% in the MSP group and 3.20% in the NRG_H group, which were 9.08-fold and 12.8-fold higher than the 0.25% observed in the NC group (Figure 6D).

**Figure 7:**
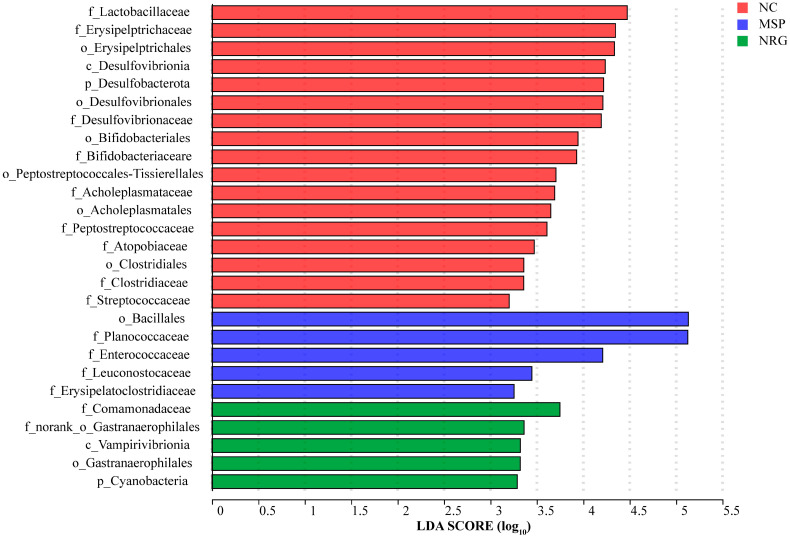
The linear discriminant analysis histogram of the control and NRG_H groups. Each experimental group consisted of five mice.

In the LFEse multi-level species differential analysis, it was found that Comamonadaceae, Vampirivibrionia, Cyanobacteria, and Gastranaerophilales were enriched characteristic bacteria after the NRG_H treatment (Figure 7).

## 4. Discussion

This investigation initially elucidated that NRG, administered at high, medium, and low concentrations, uniformly accelerated gastric emptying and small intestinal propulsion in healthy mice, with the high dose exhibiting the most pronounced effect. Jeong et al.‘s research findings suggest that the primary metabolites of flavonoids are glucuronides and sulfates [18]. In selecting an appropriate animal model for NRG administration, mice were deemed more suitable than rats as the latter excrete minimal quantities of sulfates, particularly at lower concentrations. Conversely, the concentration of sulfates in mice closely mirrors that observed in human plasma and urine.

This research indicated that the enhancement of gastrointestinal motility by naringenin in mice was primarily accomplished via three mechanisms which are discussed below.

### 4.1. Regulating the Secretion of Related Hormones

MTL and GAS are commonly referred to as “accelerator hormones”, whereas VIP is classified as an “inhibitory hormone” [19]. The impact of NRG_H on the regulation of these three gastrointestinal hormones was assessed across various doses. The results indicated that all NRG-treated groups positively modulated the levels of the first two hormones—MTL and GAS—which was consistent with their observed effects in past mice constipation models [14]. Through in vitro experiments, it was also found that NRG could activate the Ghrelin receptor [20] and affect the secretion of CCK [21].

### 4.2. Improving the Expression Levels of Proteins Associated with the SCF/c-kit Signaling Pathway

The SCF/c-Kit system forms a classical canonical ligand/receptor tyrosine kinase signaling transduction pathway that orchestrates smooth muscle contraction. The interstitial cells of Cajal (ICC) act as specialized gastrointestinal pacemaker cells [22,23], where they serve a pivotal role in gastrointestinal motility and gastric emptying [24,25]. The quantity and morphological structure abnormalities of ICC directly affect gastrointestinal motility [26]. Functionally, ICCs act as “pacemaker” cells, generating spontaneous slow waves to modulate gastrointestinal motility [27]. The growth, phenotype maintenance, and functional activities of ICCs are governed by the SCF/c-Kit signaling pathway, which comprises the specific receptor c-Kit (a tyrosine kinase receptor) and its natural ligand, SCF. This pathway governs the cellular proliferation, differentiation, and migration of ICCs [28,29]. Furthermore, the SCF/c-Kit pathway enhances the sensitivity of gastrointestinal hormones [30]. Flavonoids, such as nobiletin and quercetin, can modulate this signaling pathway to exert their effects [31,32]. The present study revealed that NRG upregulates the levels of SCF and c-Kit proteins in the stomach, duodenum, and colon, suggesting that one mechanism by which NRG promotes gastrointestinal motility is through the induction of increased ICCs via the SCF/c-Kit pathway.

### 4.3. Influencing the Composition of Gut Microbiota

NRG exhibits a multifaceted regulatory impact on gut microbiota, encompassing both compositional and metabolic aspects, as follows: (1) It regulates gut microbiota composition, as NRG has been shown to influence the composition of gut microbiota, particularly in polycystic ovary syndrome, where it enhances the diminished prevalence of *Prevotella and Gemella* while concurrently increasing the presence of *Butyricimonas*, *Lachnospira*, *Parabacteroides*, *Butyricicoccus*, *Streptococcus*, and *Coprococcus*. Among these, the genera *Butyricicoccus*, *Roseburia*, and *Streptococcus* can directly or indirectly enhance the production of short-chain fatty acids (SCFAs) like acetate, butyrate, and propionate [33]. These chemicals serve as potential biomarkers and therapeutic targets indicative of metabolic health, exerting a multifaceted influence on a host’s metabolism. They function as a conduit of communication between the gut and various peripheral tissues, including adipocytes, intestinal cells, and endocrine cells, thereby modulating appetite, energy expenditure, lipid metabolism, and insulin sensitivity, as well as influencing intestinal barrier function. *Butyricicoccus pullicaecorum* enhances butyrate production by activating SCFA transporters and/or receptors, which may prevent or ameliorate the clinical manifestations of colorectal cancer [34]. *Roseburia* species generate SCFAs through the fermentation of indigestible carbohydrates in the gut, potentially providing therapeutic benefits for individuals with inflammatory bowel disease [35]. *Streptococcus* spp. produce acetate via the Wood–Ljungdahl pathway and pyruvate’s decarboxylation to acetyl-CoA. When combined with *Lactobacillus* and *Bifidobacterium* in specific proportions, these bacteria demonstrate the safe and effective alleviation of symptoms in patients with ulcerative colitis [36]. In a model where obesity is triggered by a diet rich in fats, NRG demonstrates prebiotic-like actions by adjusting the makeup of gut microbiota, which, in turn, aids in maintaining the integrity of the intestinal barrier. Furthermore, NRG augments the expression of zonula occludens-2 and tight junction proteins, thereby strengthening the integrity of the tight junctions (TJ) barrier in Caco-2 cells [37], and by preventing the movement of inflammatory agents (such as tumor necrosis factor-α, interleukin-6, and the inflammatory marker F4/80) along the gut-liver pathway, it consequently alleviates the inflammatory conditions in the intestines of obese animals in animal models [38]. (2) The regulation of the growth and gene expression patterns of gut symbiotic microorganisms is also supported by Jenni Firrman’s research findings, which revealed that NRG exhibits differential effects on the growth and gene expression patterns of gut symbiotic microorganisms, including *Ruminococcus gauvreauii*, *Bifidobacterium catenulatum*, and *Enterococcus caccae*. The growth curve analysis indicated that NRG had no effect on *Ruminococcus gauvreauii*, slightly promoted the growth of *Bifidobacterium catenulatum*, and significantly inhibited the growth of *Enterococcus caccae*. In *Ruminococcus gauvreauii*, genes associated with iron absorption, such as iron transporter FeoA, were upregulated. In *Bifidobacterium catenulatum*, genes related to cellular metabolism, DNA repair, and molecular transport, including putative lipoprotein signal peptidase, ATP synthase epsilon subunit, phosphopantetheine adenylyltransferase, and holliday junction DNA helicase RuvA, were upregulated, while genes associated with thymidine biosynthesis and metabolism, for example, deoxyuridine 5′-triphosphate nucleotidohydrolase, carbohydrate kinase, and l-ribulose-5-phosphate 4-epimerase, were downregulated. For *Enterococcus caccae*, NRG enhanced the expression of the pathways involved in transcription and protein transport, and it concurrently suppressed the expression of genes linked to sugar transport and purine synthesis [39]. Additionally, the existing literature indicates that *Turicibacter*, *Alistipes*, *Lachnospiraceae_NK4A136_group*, and *Ruminococcus* are negatively correlated with the expression of SCF/c-Kit-related proteins [40]. Consistent with these findings, our study demonstrated that NRG promotes the expression of SCF and c-Kit proteins while concurrently reducing the relative abundance of *Lachnospiraceae_NK4A136_group*. This alignment with previous research underscores the potential mechanistic pathways through which NRG may exert its therapeutic effects.

Among common dietary polyphenols, NRG exhibits the most pronounced impact on the growth of probiotics such as *Lactobacillus rhamnosus* and commensal bacteria like *Escherichia coli*, as well as pathogenic bacteria, including *Staphylococcus aureus* and *Salmonella typhimurium* [41]. This study further elucidates NRG’s significant regulatory effects on *Staphylococcus* and *Lactobacillus*. Additionally, Planococcaceae influence blood sugar levels by modulating valine production; *Bacteroides acidifaciens*, a probiotic with anti-obesity effects, enhances IgA production, thereby improving gut immune function [42,43]; Gastranaerophilales represents a beneficial gut bacterium [44]; and *Clostridia_UCG-014*, a probiotic associated with tryptophan metabolism, regulates gut homeostasis [45]. NRG positively regulates these beneficial bacteria, underscoring its multifaceted role in gut microbiota modulation.

## 5. Conclusions

NRG, when present in concentrations spanning from 6.25 mg/mL to 25 mg/mL, facilitates gastric emptying and small intestinal propulsion through the modulation of gastrointestinal muscle tissue contractions and the regulation of hormone expression levels pertinent to the digestive system, including MLT, GAS and VIP, with the highest concentration of 25 mg/mL showing the most significant effect. The underlying mechanism of action involves the upregulation of the SCF/c-Kit pathway, which augments sensitivity to gastrointestinal hormones. This effect is concomitant with an enhancement in the abundance of beneficial bacterial (e.g., Planococcaceae, *Bacteroides acidifaciens*, and *Clostridia_UCG-014*) populations.

## Data Availability

The original contributions presented in the study are included in the article, further inquiries can be directed to the corresponding author.
